# Development of an outcome measurement system for service planning for children and youth with special needs

**DOI:** 10.1111/j.1365-2214.2012.01409.x

**Published:** 2012-07-30

**Authors:** M K Kertoy, D J Russell, P Rosenbaum, S Jaffer, M Law, D McCauley, J W Gorter

**Affiliations:** * Western UniversityLondon, Ontario, Canada; † CanChild Centre for Childhood Disability Research, McMaster UniversityHamilton, Ontario, Canada; ‡ Department of Pediatrics, McMaster UniversityHamilton, Ontario, Canada

**Keywords:** child disability, measurement, outcomes, parent report, service delivery

## Abstract

*Aim* This study described the process used in developing an outcome measurement framework for system planning to improve services for children and youth with special needs and their families in a Canadian province. The study reports the results of several parent-completed measures, which would be useful in service planning as well as the acceptability and utility of these measures for use by families and service centres.

*Methods/results* Development of a theoretical framework, consultation with key stakeholders, testing the utility of selected outcome measures and initial dissemination of results were critical elements in the successful development of an outcome system. Consultation with stakeholders confirmed use of the International Classification of Functioning, Disability and Health and the child-within-family-within community model as theoretical frameworks while building valuable partnerships and identifying potential barriers to implementation. Pilot testing showed three outcome measures were feasible for families to complete and the measures provided information about services for children that was valuable to families as well as service providers. Gaps in service delivery were identified and the need for better communication between service providers and communities to facilitate integrated services was highlighted.

*Conclusion* The findings from this study can be used to implement an outcome measurement system for children with special needs and may serve as a resource for international researchers who are working to develop valid tools as well as outcome systems that are useful for system planning.

## Background

Measurement of treatment outcomes for children with special needs has advanced considerably over the past two decades (Law 13; Majnemer & Mazer 15). The most notable influence on the development and selection of outcome measures has been the worldwide adoption of the World Health Organization's International Classification of Functioning, Disability and Health (ICF) into treatment paradigms for children (World Health Organization 21). A paradigm shift in rehabilitation based on the ICF and ICF for children and youth (ICF-CY; World Health Organization 21, 22) has created the need for outcome tools that measure activity, participation and environmental factors along with assessment of impairment of body functioning (Helders *et al*. 9; Rosenbaum & Stewart 16). Measures based on multiple components of the ICF have been used to document the effectiveness of specific medical procedures (Boyd & Hays 2; Bedell 1; Thomas-Stonell *et al*. 20) or responsiveness of measures to changes because of rehabilitation programmes (Summers *et al*. 18; Wright *et al*. 24).

While researchers and clinicians now use measures to better understand the everyday experiences of children with special needs and what is important to them (Coster & Haley 5; Butler 4), most measures are being used to monitor children's progress in treatment. Measurement of outcomes about the service delivery system as a whole is not taking place. Summers and colleagues (19) demonstrated how three family report measures could be used to improve the delivery of services, but they focused on the utility of the individual measures and not on the development of an outcome measurement system. One system, interRAI, has been developed to measure health outcomes in elderly adults through the use of consistent tools to measure continuity of care and inform policy decisions (Hirdes *et al*. 10). Another outcome system developed in the US measures the outcome of government-implemented programmes to improve the skills of preschoolers in specific areas of development along with family outcomes (Bailey *et al*. 9001).

The purpose of this paper was to describe the process undertaken to develop an outcome measurement system that would describe the outcomes of children and families receiving services from children's rehabilitation organizations in one Canadian province. Additional aims were to collect data from parents that could be used to improve service delivery, and to investigate the feasibility and utility of several tools that measure environmental and/or personal factors.

## Methods

The steps taken to develop an outcome measurement system are outlined in Table 1. Phase 1 included the development of a theoretical framework on which to build a system, consultation with key stakeholders and selection of measures. Phase 2 involved the pilot testing of selected measures to determine the feasibility, acceptability, and utility of the measures for families and service providers. Utility in this study was defined as the parents’ perceptions of the usefulness of the survey information to inform service planning.

**Table 1:** Components of an outcome system for children

Critical components and steps in developing a system	Key decisions made
*Phase 1: Groundwork and development*	
(A) Development of a theoretical framework	Review of various frameworks	Decision made to include framework based on ICF Ecological model of child in family in community
(B) Consultation with key partners and stakeholders	Partners and stakeholders agreed upon Purpose and goals of the system Which children will be evaluated Structure of the system and use of the ICF as a framework How information will be used How appropriate outcome measures will be selected
(C) Selection of measures to evaluate outcomes	Criteria for selecting measures Completed by parents Appropriate for collecting information about children of varying diagnoses and ages Provide useful information for planning within the current system Valid and reliable Related to ICF
*Phase II: Pilot testing of system*	
Pilot test selected measures	Determine feasibility and utility of selected measures from the environment domain of the ICF

ICF, International Classification of Functioning, Disability and Health.

### Phase 1

Two frameworks guided the development of an outcome measurement system, the World Health Organization's (21) ICF and the ecological model of child-in-family-in-community (Bronfenbrenner & Ceci 3). The ICF recognizes activities (what individuals actually do) as well as participation (one's engagement in life in meaningful ways) and environmental factors. According to the ecological model of child-in-family-in-community (Bronfenbrenner & Ceci 3), the needs and goals of families and children are equally important components of service delivery. Included within ecological models is family-centred service, a service delivery model where parents serve as key informants and decision makers in the service delivery process for their children. The theoretical framework also included designing a system that reflected a partnership between families, service providers and government agencies and that gathered information meaningful to all parties in improving services.

Input about the conceptual framework and potential measures was sought from key stakeholders including researchers in the field of childhood disability, service providers and government agencies that provide services for children in Ontario, and children and families who receive treatment services. Five meetings/focus groups and one half day workshop were organized to elicit input on the proposed outcome system. Questions were pre-circulated to participants to spur discussion (see Table 2). Recommendations and feedback were used to form a unified framework that reflected the perspectives of multiple users.

**Table 2:** Stakeholder meetings and focus groups

Meeting/focus group and representation	Questions
Researchers (*n* = 14) Medicine Physical therapy Occupational therapy Speech and language pathology Prosthetics and orthotics	Initial impression and thoughts about the proposed measurement framework and measures Will the proposed system fulfil the goal from your perspective? If not, please give advise about what is missing. Is there any information that we should consider in the future?
Policy makers (*n* = 7) Ministry of Education Ministry of Children and Youth Services (MCYS) Ministry of Health and Long Term Care	Initial impression and thoughts about the proposed measurement framework and measures Gaps in information gathering What type of data/information do you wish you could have easily accessible? Do you think this proposed system would be useful to you?
Consumer consultants (*n* = 7) Parents of children and youth with special needs Young adult with a disability	What do you think the Ministry needs to know about your child and family to make relevant decisions? Should they know about your child's activity and participation? Should they know about your family's well-being? Do you think we would be collecting the right information? Should we ask, and if so, how do we ask about your well-being as a parent caring for a child with special needs? How is it best to collect this information?
Half day workshop (*n* = 45) Ontario Association of Children's Rehabilitation Services and children's treatment centres Service providers Community care access centres Family organizations University-based academic programmes MCYS Families Federal government agencies	What resources would you need to implement this system (financial, human, technology, etc.)? What do you think about the approach of this system (using the ICF framework)? What do you think about the implementation of the approach (the types of measures specified)? What would you do differently? What problems will the MCYS have implementing this system? Can you think of any solutions for the problems identified?

ICF, International Classification of Functioning, Disability and Health.

Fourteen researchers from medicine, physical therapy, occupational therapy, speech language pathology, and prosthetics and orthotics from Australia, Canada, Finland, Germany, the Netherlands, Sweden and the USA supported the ICF as an appropriate framework for measurement and provided feedback on specific measurement tools. Seven representatives from three government ministries responsible for overseeing provision of services to children in Ontario recognized the need for collection of better data about services and the need for greater sharing of information across ministries. Families were generally supportive of the framework. Seven consumer consultants (including parents of youth and one young adult with a disability) recommended that families provide information anonymously so it could not be used to deny services. Families felt the quality of life scale should not be an outcome measure as many factors in addition to level of service determine one's quality of life. Service providers and administrators had concerns that language barriers might affect parents’ ability to complete outcome measures. They also wondered about raising unrealistic expectations for families who would not be provided services they identified as needs.

### Phase 2

**Figure 1:**
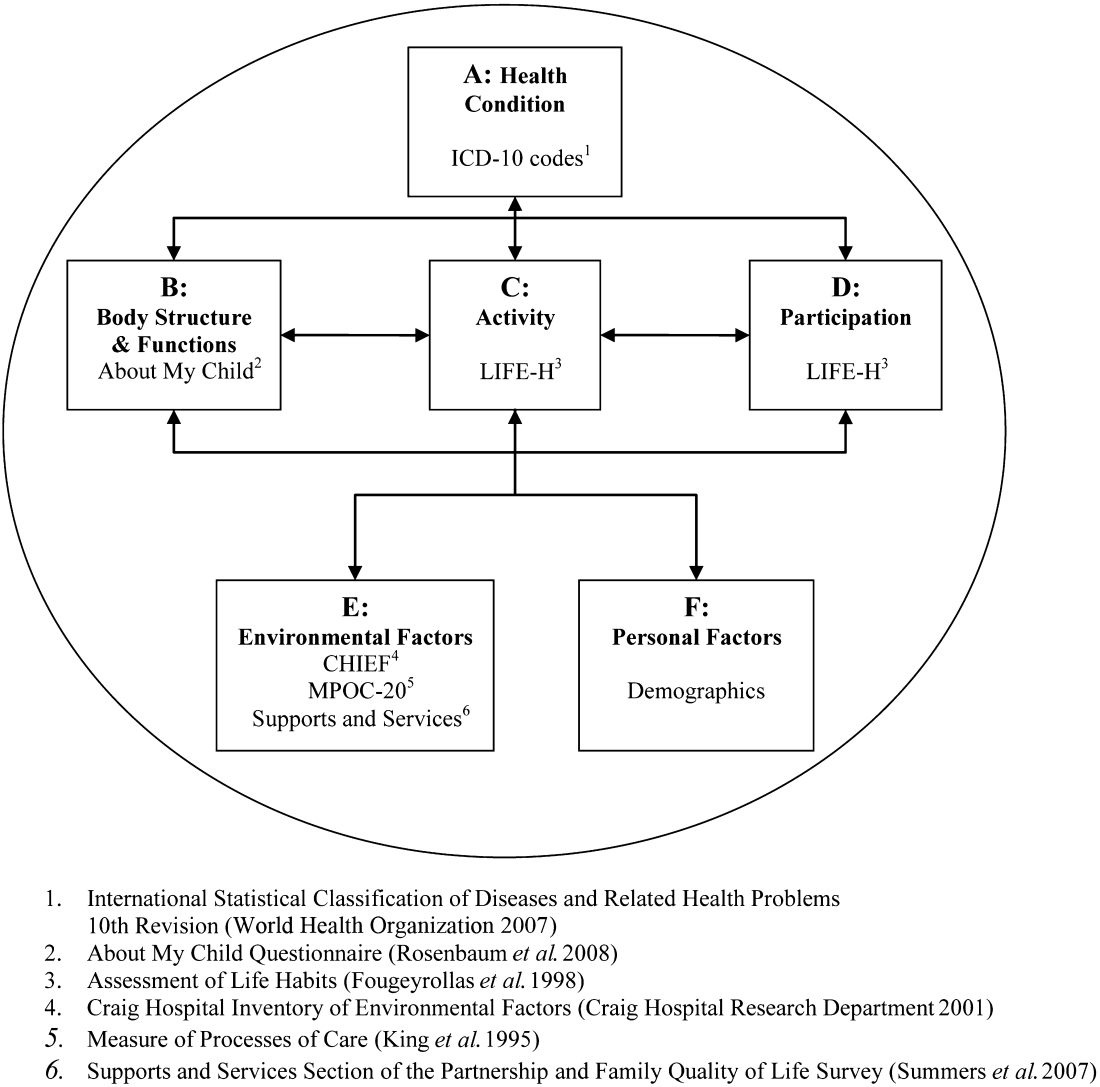
International Classification of Functioning, Disability and Health framework with selected outcome measures.

To test the utility of the data, three measures were identified within the environmental and personal factors domain of the ICF and pilot tested with families (see Fig. 1). The measures covered these environmental factors: parents’ perception of the family-centredness of services; environmental barriers to participation; and service needs and gaps. Demographic information about the child and family was also collected. The package of measures contained a total of 89 close-ended questions and took on average 24 min to complete. Furthermore, the measures were suitable for (or modified for use with) children, allowed parents to give their perspective, and provided information at the system level to improve service delivery. Administrators were interviewed and provided information about the treatment centres’ perspectives of the utility of the measures.

The Measure of Processes of Care (MPOC-20; King *et al*. 11) assesses parents’ perceptions of services and helps service providers to understand how family-centred families perceive the service system to be. The degree to which families perceive services to be family-centred is associated with overall satisfaction with services and with better parent mental health (King *et al*. 12). The MPOC-20 exhibits excellent reliability and validity for a short, self-administered measure. The internal consistency coefficients ranged from 0.77 to 0.87 for the scales and test–retest coefficients for the scales range from 0.81 to 0.86.

The Craig Hospital Inventory of Environmental Factors (CHIEF; Craig Hospital Research Department 6) measures environmental barriers within the domains of school/work, physical environment, policies, services, and attitudes and supports. The 25-item CHIEF has been used as a proxy measure by parents reporting on the barriers their children with disabilities encountered at home and in the community (Law *et al*. 14).

The Supports and Services inventory of the Partnership and Family Quality of Life (Supports and Services Questionnaire; Summers *et al*. 18) is an assessment of the services children and families receive and their perception of the adequacy of these services to meet the child's and families needs. A total of 28 services are listed, categorized by services specific to the child (e.g. therapies, service co-ordination, specialized equipment, etc.) and services specific to the family (e.g. resources, provision of care, legal rights, etc.). Parents are asked if the service listed is needed, if the response is yes, they are then asked how much of the service is received (none, some but not enough, and enough). Reliability coefficients are not available for the Supports and Services Questionnaire as the service inventory will vary from family to family and therefore the items do not logically cluster together (email communication with Jean Summers on 16 October 2008).

### Procedures

Ethics approval was obtained from the Ethics Review Board at McMaster University. Three of 19 children's treatment centres in Ontario were approached and agreed to participate in this pilot study. These centres varied by geographic location (Northern, Central East and Central West), caseload size (ranging from approximately 1000 to 4000+ clients) and number of satellite sites (three to four satellite sites each). Each centre created a client list of families caring for children with special needs between birth and 16 years of age who were on the active caseload at the centre and randomly selected the sample from this list. The list was kept at the centre and not shared with the investigators. Packages containing the study materials for each family were collated at *CanChild* Centre for Childhood Disability Research and delivered to each treatment centre where the address labels were affixed and mailed. Each package contained a letter from the centre administrator introducing the study, a letter outlining the purpose of the study, the measures, a feedback form about the clarity and usefulness of each measure, and a stamped, addressed reply envelope, which would go directly to *CanChild*. The treatment centres mailed research packages to a total of 617 families in October and November 2007. Centres sent pre-printed reminder postcards to each family 2 weeks after the initial mailing. One hundred and eighty-two families returned the completed packages (with anonymity preserved) directly to our research centre in the reply envelope for a response rate of 29.5%. Table 3 presents demographic information about the families who participated.

**Table 3:** Demographics of the study sample

Demographic	*n* (%)
Child's age	
Less than 5 years of age	108 (59.3)
5 years of age and older	72 (39.6)
Missing	2 (1.1)
Child's sex	
Male	120 (65.9)
Female	60 (33.0)
Missing	2 (1.1)
Type of community	
Pop. 250 000+	29 (15.9)
Pop. 50 000–249 999	88 (48.4)
Pop. 20 000–49 999	4 (2.2)
Pop. 2500–19 999	26 (14.3)
Pop. < 2500	24 (13.2)
Missing	11 (6.0)
Two parent household	
Two parent family	154 (84.6)
Single parent family	28 (15.4)
Parent's income	
Less than $29 999	33 (18.1)
$30 000–$59 999	43 (23.6)
$60 000–$89 999	53 (29.1)
More than $90 000	46 (25.3)
Missing	7 (3.8)

The families’ responses to the measures as well as their perspectives on the utility of the measures were entered into a database at *CanChild* and analysed using Statistical Package for the Social Sciences (SPSS, Version 16; SPSS, Inc., Chicago, IL, USA). Customized reports were prepared with summarized results for each measure and individualized for each of the centres. Three weeks after the study report was mailed to the centres, telephone interviews were arranged with an administrator or programme manager from each centre to discuss the usefulness of the data and to seek feedback about the data collection process.

## Results

### Appropriateness and utility: family perspective

Families were most efficient in completing the MPOC-20 with 95% of families providing full information. Seventy-two per cent of families provided full information on the Supports and Services Questionnaire. The CHIEF was the most challenging measure for families to complete with 62% of parents completing the entire measure. Several families with children under 5 years old identified that the CHIEF questions were not as relevant for their children because the families were usually with their children and helped them overcome many of the potential barriers.

The families rated the utility of the MPOC-20, CHIEF, and the Supports and Services section of the Partnership and Family Quality of Life Questionnaire highly. Over 75% of families indicated that they somewhat agreed to strongly agreed (a rating of between 3 and 5 on a five-point scale) on the utility of each of the three measures (81% MPOC, 76% CHIEF and 90% Supports and Services).

### Families’ responses to measures

**Figure 2:**
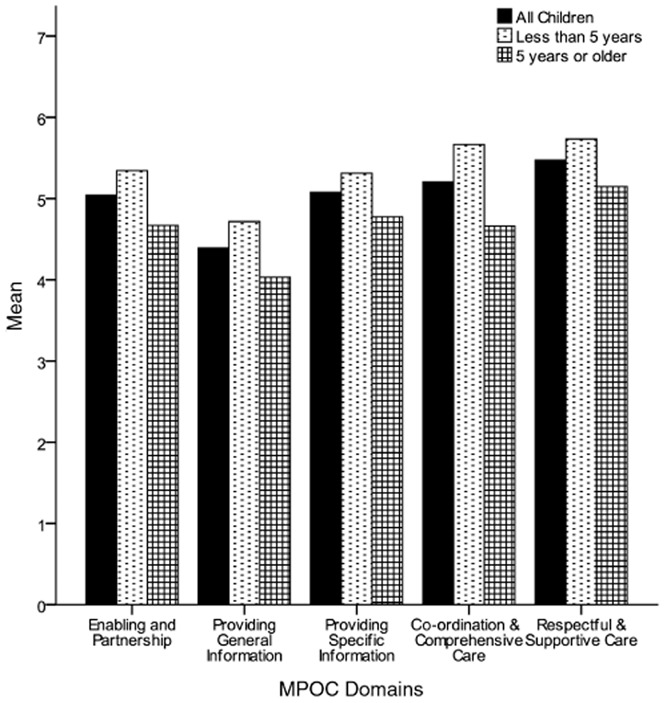
Parents’ perceptions of family-centred service delivery using the Measure of Processes of Care (MPOC)-20.

The scores for all parents on the MPOC-20 were relatively high indicating families believed that the services they received were family centred (see Fig. 2). Parents’ ratings were highest for the Respectful and Supportive Care domain (M = 5.47, SD = 1.30) and lowest for the Providing General Information domain (M = 4.39, SD = 1.59). A general linear model with repeated measures showed that there were significant differences between the five domains (*F* (1, 156) = 2335, *P* < 0.0001). Results of paired sample *t*-tests indicated that the scores for Providing General Information were significantly lower and Respectful and Supportive Care were significantly higher from the other four domain scores.

Two groups of parents of children of different ages were created to determine if their ratings of family centred services were influenced by the age of their children. As seen in Fig. 2, parents of children five and older rated all five domains of service delivery slightly lower (domain means ranged from 4.03 for Providing General Information to 5.15 for Respectful and Supportive Care) than parents of children younger than 5 years (domain means ranged from 4.72 for Providing General Information to 5.73 for Respectful and Supportive Care). Parents of children five and older rated the Co-ordination and Comprehensive Care domain much lower than parents of children less than five (there was the largest gap in ratings for this domain, M = 4.66 for parents of children 5 years and older versus M = 5.67 for parents of children less than 5 years). *T*-tests were used to test the differences between the mean ratings by parents of children older than 5 years versus those by parents of children under five on each of the five domains. Differences in ratings by the two groups of parents were significant at the 0.05 level for all domains.

**Figure 3:**
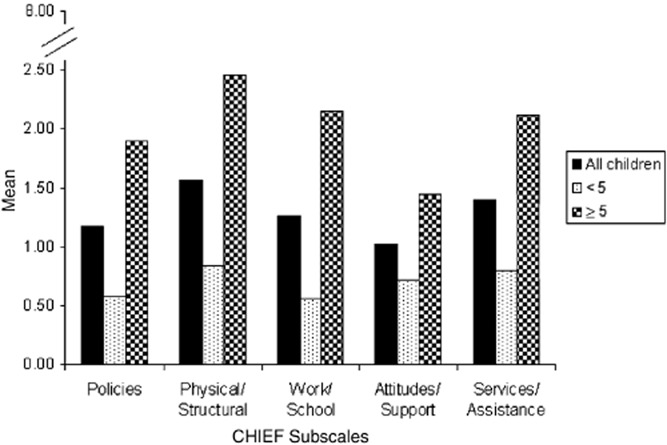
Craig Hospital Inventory of Environmental Factors (CHIEF).

Figure 3 shows the mean frequency magnitude product scores of parents’ perceived barriers for five environmental subscales of the CHIEF. In descending order of impact, parents reported that their children encountered the highest perceived barriers to Physical and Structural (M = 1.56, SD = 1.95), followed by Services and Assistances (M = 1.39, SD = 1.66), Work and School (M = 1.26, SD = 1.99). Policies (M = 1.17, SD = 1.72) and Attitudes and Supports (M = 1.02, SD = 1.68). A general linear model with repeated measures indicated a significant difference among subscale scores (*F* (1, 152) = 123, *P* < 0.001). Paired sample *t*-tests indicated that the Attitudes and Supports subscale was significantly lower than the Physical and Structural and Services and Assistance subscales.

Figure 3 also displays the mean frequency magnitude product scores of two parent groups, those with children age five and older and those with children younger than 5 years of age. The parents of children who were 5 years or older perceived their children to encounter greater barriers in each of the five environmental scales (means ranged from 1.44 to 2.45 across the five contexts) than parents of children younger than 5 years (means ranged from 0.55 to 0.84 across the five contexts). *T*-tests were conducted between the scores for the two groups of parents and indicated that these differences were significant for all five environmental scales (*P* < 0.001).

**Figure 4:**
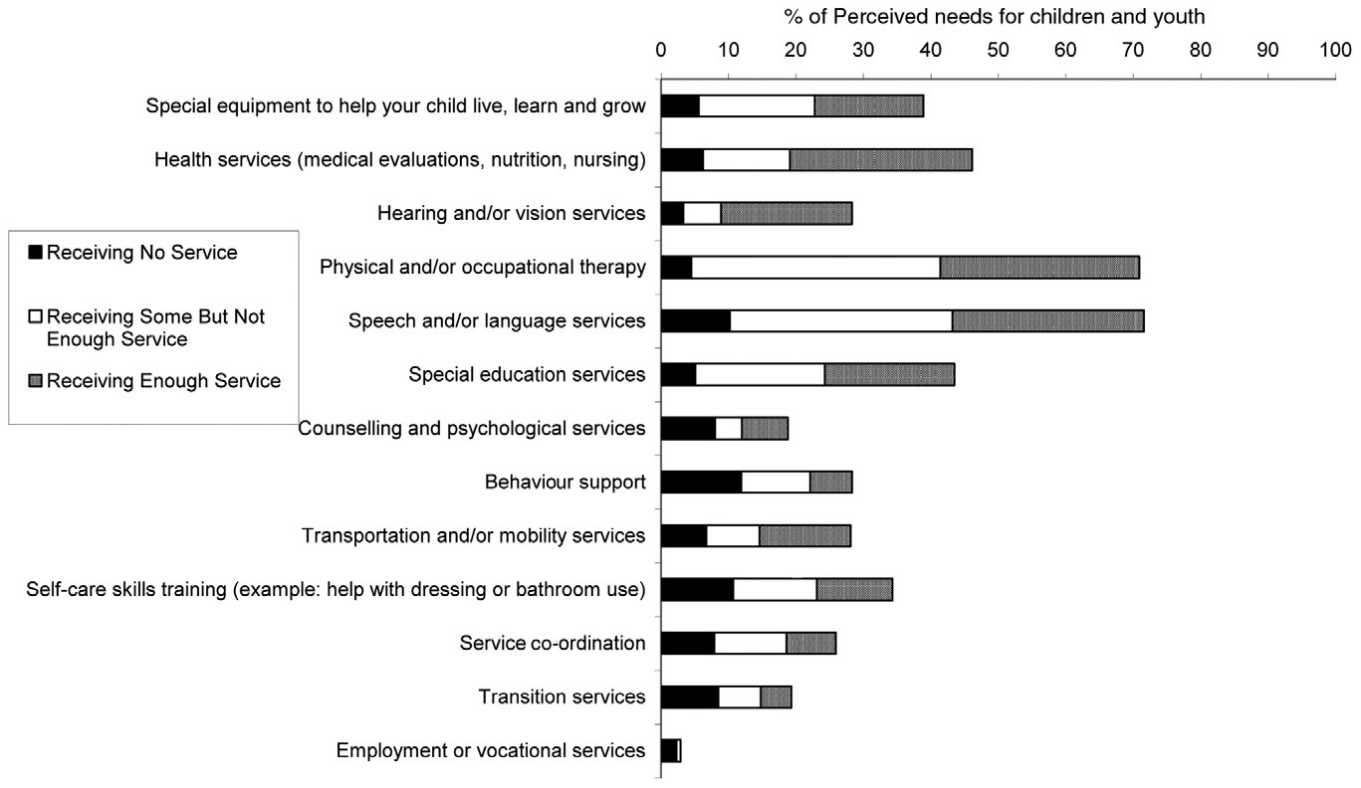
Children and youth needs. Parental perception of services needed and amount needed from the Supports and Services Questionnaire.

**Figure 5:**
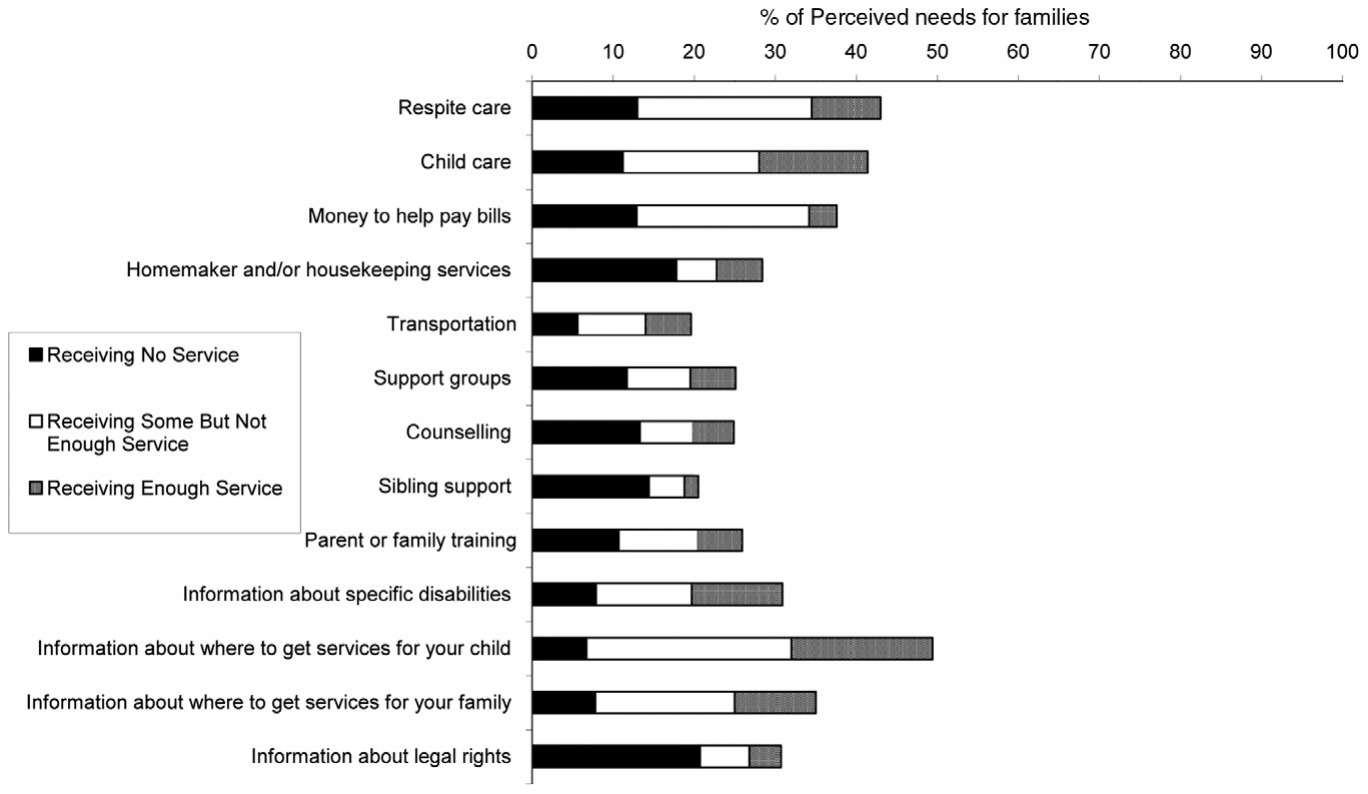
Family needs. Parental perception of services needed and amount needed from the Supports and Services Questionnaire.

The bars on Figs 4 and 5 reflect the percentage of families who identified each service as a need. The designs within each bar reflect how much service the families perceive they are receiving (no service, some, but not enough service, or enough service). The five services perceived to be most needed for children/youth were: speech and language therapy (72%), physical and occupational therapy (72%), health services (47%), special education (44%) and use of adaptive equipment (39%) (Fig. 4).

The five services perceived to be most needed by families were: Information about where to get services for their child (50%), Respite Care (44%), Child Care (42%), Money to help pay bills (38%) and Information about where to get services for the family (35%) (refer to Fig. 5). The largest unmet needs (defined as the per cent of families who say the family needs the service and the family is receiving some or no service) for families occurred in the following service areas: Sibling support (21% of families report the need; 92% of these families report it as an unmet need), Information about legal rights (31% need; 87% of these families report unmet need), Homemaking services (28% need; 80% of these families report unmet need), Parent or Family Training (26% need; 78% of these families report unmet need) and Respite Care (44% need; 80% of these families report unmet need).

### Appropriateness and utility: administrators’ perspective

Each treatment centre was sent a summary of the results for their centre compared with the cumulative responses from all three centres. Teleconferences were arranged with administrators from each centre once they had time to read and discuss the centre reports with colleagues. All administrators supported the use of all three measures and identified how information gathered could be useful in improving service delivery for families. Administrators felt strongly that while the information collected was very useful, care needed to be taken to ensure the data were interpreted within a local context.

Scores on the MPOC-20 have been or will be used in the future to prioritize areas of service needs at each treatment centre. For example, providing general information to families was the lowest scoring domain on the MPOC-20. Centres might wish to survey their families about what types of information are helpful and then facilitate easy access to the information through developing new resources or linking up with existing resources in the community or on-line. If the need for information was a province-wide need, then it may be an opportunity for government and centres to work together to identify strategies to support families in finding the information they need.

The CHIEF was viewed as helping to validate barriers that had previously been reported to the centres by families. Information from the CHIEF was also seen as useful for children's treatment centres to begin to talk with community partners about how to work together to minimize barriers for families, as some families may receive services in multiple locations outside treatment centres.

The Supports and Services Questionnaire was seen as useful for helping to identify unmet needs of children and families. Families and service providers could discuss ways to remediate unmet needs. Additional services may be provided by children's treatment centres or families could be directed to services available in the community. Information from the measures could highlight inequities in accessing services, which would be important for government decision makers in planning future services. Information from both the CHIEF and the Supports and Services Questionnaire highlighted the need for ongoing discussion of service needs between families, centres, communities, and government.

## Discussion

This study described the process used in developing an outcome measurement framework for system planning to improve services for children and youth with special needs and their families and the modification and pilot testing of selected measures for use with children and families. Development of outcome systems like the one described here that can help identify strengths and needs of service delivery systems are critically needed. An important first step was to be guided by a conceptual framework. The process of gaining feedback from stakeholders affirmed use of the ICF as a framework and identified a number of measures that were potentially useful for measuring outcomes at a service system level. The process of including parents, service providers, administrators and government agencies in the planning process was critical for gaining support for the use of an outcome system.

Knowledge exchange activities with all stakeholders during the planning stages of the outcome measurement system provided valuable input and helped to build partnerships and trust. From our experience, both families and service providers were happy to be included in the process even though they expressed initial concerns about how the information would be used. This opened up opportunities to discuss their concerns and gave us a sense of what barriers might occur if we had tried to implement the system without this crucial step.

The pilot testing showed that the measures were feasible to administer to families through a mailed survey, but the process could be improved. The response rate was just under 30% by employing one mailing and a follow-up reminder. Strategies to increase the response rate would be warranted. The use of online surveys and clearer instructions about the services for which parents needed to provide feedback could improve response rates and accuracy of responses.

The information gained from three pilot measures was perceived as useful to both families and service organizations. The MPOC and the CHIEF showed variation across individual scales within each measure as well as variation with age of the children. Parents of children 5 years and older perceived the services they received to be less family centred on the Co-ordinated and Comprehensive Care domain of MPOC than parents of children younger than 5. Similarly, parents reported greater barriers as measured by the CHIEF as their children got older. As parents indicated some challenges in completing the 25-item CHIEF (refer to section on Appropriateness and Utility), recent work has identified 10 barriers that parents identified most frequently and were used to develop the CHIEF for Children–Parents Version with the authors’ permission. Pilot testing with 45 families on the CHIEF for Children–Parent Version showed acceptable internal consistency (0.76) and test–retest reliability (0.73) (McCauley *et al*.^2012^). The responsiveness of these measures to family's perceptions of changes in service delivery supports their use as outcome measures to monitor perceptions about service delivery longitudinally.

There was a need to interpret the findings from these measures within a local context. The three children's treatment centres who participated in this study did not provide the same services in the same way. By using local information, service providers and policy makers can determine if there is a genuine gap in a particular service or if families are unaware that the service is available. Service delivery might be improved through better co-ordination and planning of services, through improved marketing of the service, or through greater community outreach to meet the needs of families. The most effective approach to improving services can be illuminated by the families and service providers involved in a community or service region.

The current study highlighted the need for integration of services and greater communication between the children's treatment centres and the communities where children are accessing other services. The families indicated that they received services from multiple locations in their community and that co-ordination of services between different service agencies was important. The outcome measurement system helped to identify gaps in the delivery of services and provided valuable information to children's treatment centres and community agencies that are in the process of building an integrated service system.

## Key messages

1. There is a need to use outcome measures to obtain information about service delivery systems for children with special needs in order to plan for future services.
2. Important elements of an outcome measurement system for children with special needs included development of a theoretical framework, consultation with stakeholders, use of feasible outcome measures and dissemination of results for use in future service planning.
3. The CHIEF for Children–Parent Version, the MPOC-20 and the Supports and Services Inventory were feasible for completion by families.
4. Three outcome measures were shown to provide valuable information about service delivery for parents and service providers.
5. Measuring outcomes at the systems level helped to identify gaps in services and the need for greater communication between service providers and community agencies.
6. Data collected need to be interpreted within the local context to ensure that information is not misinterpreted.

## Conflict of interest

The authors declare that they have no competing interests.
